# The Chicago Meeting of the American Medical Association

**Published:** 1908-07

**Authors:** F. G. Hodgson


### EDITORIALS

The Business Office of the JOURNAL-RECORD is 1 1-2 to 5 1-2 South Broad St.
The Editorial Office is 1014-15 Century Bldg.
Address all Business Communications to Journal-Record of Medicine, 1 1-2 to 5 1-2 South Broad Street.
Make remittances payable to THE JOURNAL-RECORD OF MEDICINE.
On matters pertaining to the Editorial and Original communications, address Edgar G. Ballenger, M. D., Atlanta, Ga.
Reprints of original articles will be furnished at cost price. Requests for the same should always be made in THE MANUSCRIPT.
We will present, postpaid, on request, to each contributor of an original article, twenty (20) marked copies of THE JOURNAL-RECORD OF MEDICINE containing such article.

### THE CHICAGO MEETING OF THE AMERICAN MEDICAL ASSOCIATION.

The fifty-ninth annual session of the American Medical Association which was held in Chicago, June 2-5, 1908, was perhaps, the largest gathering of physicians that has ever occurred in this country. There were about 6,500 doctors present, from all parts of the world; every state in the Union had its representatives; they came from Canada, Mexico, all parts of Europe, also from China and Siam. Among the distinguished foreign guests, were Dr. Schafer, Edinburg; Drs. Collins and Beevor, of London; Drs. Sauerbruch, Plannenstiel; Jansen and Martin, of Germany.

The papers read in each section were by the best men in their special branches. The paper of President Burrell, of Boston, on, "A New Duty of the Medical Profession: The Education of the Public in Scientific Medicine;" and the Oration on Medicine by Dr. Thayer, of Baltimore, on "The Relations of the Physician to the Public," should be read by every practitioner. The paper, however, which impressed me most of all was the address on Surgery, "The Cancer Problem," by Dr. Crile, of Cleveland. This was very forcefully and impressively delivered, and showed that he was a thorough master of his subject.

The different sections were very well attended. The symposium on, "Caesarian Section," in the section of Obstetrics and Gynaecology," brought out some excellent papers. Dr. Pfannenstiel, of Germany, read one; Dr. McPherson, of New York, repeated 186 cases operated upon at the New York Lying In Hospital.

The scientific exhibit was of a very high order; many specimens were shown, not only by hospitals and medical schools, but also by individuals. The commercial exhibit was also most interesting.

The House of Delegates did their work promptly and in a most business like manner. There was perfect harmony, and all seemed to be working for the very best interest of the association. Every one was given a hearing and treated with utmost courtesy and consideration. If there was any political ring at work, it was very carefully concealed.

The entertainments were numerous and attended by large crowds. Chicago furnished accommodations for this large number of physicians without undue crowding. The city has many points of interest and every one seemed to be enjoying himself and commented upon the fact that he was glad he had come.

The next session will be held in Atlantic City. This place was again chosen because it is one of the few places that can furnish accommodations and amusement for so large a crowd. Col. Gorgas was unanimously elected president for next year and this was considered a wise and happy selection by all.

F. G. HODGSON.

